# Erratum: Augmented transcutaneous stimulation using an injectable electrode: a computational study

**DOI:** 10.3389/fbioe.2024.1431976

**Published:** 2024-06-03

**Authors:** Frontiers Production Office

**Affiliations:** Frontiers Media SA, Lausanne, Switzerland

**Keywords:** transcutaneous electrical nerve stimulation, neuromodulation, electrical stimulation (EStim), vagus nerve stimulation, electrode technology, selective stimulation of deep nerves, FEM, neuron simulation

Due to a production error, there was a mistake in the legend for **Figure 7** as published. The caption for **Figure 7C** was written incorrectly as “(C) Box plot of activation thresholds of all on-target Aβ vagal fibers compared to all off-target Aβ cutanoues fibers responsible for paresthesia and off-target Aβ cutaneous fibers responsible for noxious sensations.”

The correct legend appears below.

“FIGURE 7 | **(A)** Full FEM model used in the biophysical study. The 1 kΩ resistor between the two collectors in the simplified transcutaneous coupling FEM model was replaced with the vagus nerve, Injectrode connections down to the vagus nerve, and Injectrode interfaces with the vagus nerve. This subfigure is reproduced from Figure 2C. **(B)** Box plot of all axons showing that the Injectrode system reduced the current required to activate Aβ vagal fibers by more than an order of magnitude compared to using only surface electrodes. This large difference was seen across TES patches of different sizes. **(C)** Box plot of activation thresholds of all on-target Aβ vagal fibers compared to all off-target Aβ cutanoues fibers responsible for paresthesia and off-target A δ cutaneous fibers responsible for noxious sensations. **(D)** Investigating the effect of TES patch side length on cutaneous and vagus Aβ-fiber activation. Increasing the TES patch side length increasedmedian thresholds, and this effect was less pronounced in vagal Aβ fibers, improving the ratio of Aβ vagal activation to Aβ cutaneous activation. The Injectrode system achieved preferential on-target activation one to two orders of magnitude better than using surface stimulation electrodes alone.”

The publisher apologizes for this mistake.

